# The Honeybee Gut Microbiota Is Altered after Chronic Exposure to Different Families of Insecticides and Infection by *Nosema ceranae*

**DOI:** 10.1264/jsme2.ME18169

**Published:** 2019-08-03

**Authors:** Régis Rouzé, Anne Moné, Frédéric Delbac, Luc Belzunces, Nicolas Blot

**Affiliations:** 1 Université Clermont Auvergne, CNRS, Laboratoire “Microorganismes: Génome et Environnement” F-63000 Clermont–Ferrand France; 2 INRA, Laboratoire de Toxicologie Environnementale UR 406 A&E, CS 40509, 84914 Avignon France.

**Keywords:** honeybee microbiota, insecticides, parasite, dysbiosis

## Abstract

The gut of the European honeybee *Apis mellifera* is the site of exposure to multiple stressors, such as pathogens and ingested chemicals. Therefore, the gut microbiota, which contributes to host homeostasis, may be altered by these stressors. The abundance of major bacterial taxa in the gut was evaluated in response to infection with the intestinal parasite *Nosema ceranae* or chronic exposure to low doses of the neurotoxic insecticides coumaphos, fipronil, thiamethoxam, and imidacloprid. Experiments were performed under laboratory conditions on adult workers collected from hives in February (winter bees) and July (summer bees) and revealed season-dependent changes in the bacterial community composition. *N. ceranae* and a lethal fipronil treatment increased the relative abundance of both *Gilliamella apicola* and *Snodgrassella alvi* in surviving winter honeybees. The parasite and a sublethal exposure to all insecticides decreased the abundance of *Bifidobacterium* spp. and *Lactobacillus* spp. regardless of the season. The similar effects induced by insecticides belonging to distinct molecular families suggested a shared and indirect mode of action on the gut microbiota, possibly through aspecific alterations in gut homeostasis. These results demonstrate that infection and chronic exposure to low concentrations of insecticides may affect the honeybee holobiont.

Insects are exposed to many stressors in their environment, particularly their gut since it is both the site of development of many pathogens and the site of exposure to ingested xenobiotics. At the interface between the environment and the insect, the gut microbiota is sensitive to biotic and abiotic stressors. The consequences for the host of dysbiosis may be pleiotropic because the microbiota is connected to the physiologies of the gut and nervous and immune systems (56, 71).

The environmental stressors of the European honeybee (*Apis mellifera*) have attracted interest due to the ecological and economical importance of this pollinator as well as the colony losses that have been observed worldwide over the last few decades (19, 79). The origin of these losses is assumed to be multicausal, involving pathogens, such as the highly prevalent gut parasite *Nosema ceranae*, and agrochemical contaminants, particularly neurotoxic insecticides (34, 39).

*N. ceranae* is a fungal-related parasite belonging to *Microsporidia*. This obligate intracellular parasite proliferates within the epithelial cells of the adult honeybee midgut and affects metabolic, digestive, and immune functions as well as the gut epithelium renewal rate (5, 39, 64). Consequently, *N. ceranae* may reduce the honeybee lifespan and provoke colony depopulation.

Honeybees are also exposed to a number of pesticides through the foraging of contaminated pollen and nectar that are brought back to the colony (10). Some chemicals are deliberately introduced into hives by beekeepers, particularly the acaricides used against the ectoparasite *Varroa destructor*. These pesticides may be detected in hive matrices, including wax, pollen, and honey (8, 18, 51, 62). Therefore, all colony members are chronically exposed, particularly through their diet. The most abundant and ubiquitous contaminants of matrices are varroacides. Systemic insecticides commonly used in agriculture, including neonicotinoids and phenylpyrazoles, are also frequently detected in hives. These compounds are neurotoxic insecticides (14). Even at low doses, they may alter honeybee behavior, learning, and memory and induce an immune response as well as increases in detoxifying activities (9, 17, 20, 32, 33). Some of these effects, including cell damage, have also been observed in the midgut (5, 13, 15, 63).

*N. ceranae* and ingested pesticides are in contact with the honeybee gut and may alter its physiology. Therefore, the composition and function of gut microbial communities may be affected by these stressors (35, 44, 53). The worker honeybee gut core microbiota, which is shared among individuals, is composed of five major bacterial taxa comprising the *Lactobacillus* Firm-4 and *Lactobacillus* Firm-5 clades, the *Gammaproteobacteria Gilliamella apicola* and *Snodgrassella alvi*, and species related to the *Bifidobacterium asteroides* cluster (49). Other taxa, although less abundant, are also present, particularly *Alphaproteobacteria*. Most of the 10^8^ to 10^9^ intestinal bacteria are localized within the hindgut. The crop and midgut are less colonized (36). The intestinal microbiota contributes to host homeostasis. It stimulates the immune system, constitutes a biological barrier against pathogens, and participates in digestion (29, 49, 50, 52, 80). Thus, the impact of stressors on the honeybee and its gut may be explained, at least partially, by an imbalanced microbiota, *i.e*. dysbiosis.

In the present study, we investigated how the composition of the honeybee gut microbiota may be affected under laboratory conditions after an infection with *N. ceranae* or after a chronic exposure to neurotoxic insecticides belonging to three families: the phenylpyrazole fipronil, the neonicotinoids thiamethoxam and imidacloprid, and the organophosphate acaricide coumaphos. Quantitative real-time PCR (qPCR) was used to assess changes in the abundance of the major bacterial groups of the honeybee core microbiota, namely, *Lactobacillus* spp., *Bifidobacterium* spp., *Alphaproteobacteria* and *Gammaproteobacteria*, and the species *G. apicola* and *S. alvi*.

## Materials and Methods

### Honeybee artificial rearing, infection with *N. ceranae*, and chronic exposure to insecticides

Two experiments were performed using *A. mellifera* interior worker bees collected on the frames of five colonies of the same apiary at the Laboratoire Microorganismes: Génome et Environnement (Clermont-Ferrand, France) in February 2017 (winter bees) and July 2017 (summer bees). Each of the five colonies represented an independent replicate. Honeybees were anesthetized with CO_2_ and placed into groups of 70 individuals in Pain-type cages containing a 5-mm piece of PseudoQueen (Inkto Supply, Vancouver, Canada). Honeybees were maintained at 33±1°C under a humidified atmosphere and fed 50% (w/v) sucrose syrup supplemented with 1% (w/v) Provita’Bee (ATZ Diététic, Mas Cabardès, France) *ad libitum*, as previously described (4). Dead bees were removed and sucrose consumption was quantified daily.

Seven experimental groups, including an untreated control, were tested for each colony replicate. Honeybees were collectively infected using a feeder containing approximatively 150,000 spores per bee in 2.5 mL of sugar syrup that was maintained for 24 to 34 h until complete consumption. *N. ceranae* spores were obtained from previously infected honeybees and purified according to Vidau *et al.* (81). Chronic exposure to insecticides was performed by feeding non-infected honeybees with sugar syrup supplemented with either 0.25 or 1.0 μg kg^−1^ fipronil, 650 μg kg^−1^ coumaphos, 1.7 μg kg^−1^ thiamethoxam, or 3.5 μg kg^−1^ imidacloprid. Insecticide stock solutions were prepared in DMSO, leading to a final concentration of 0.05% DMSO (v/v) in the syrup. Non-intoxicated bees (*i.e*. untreated control and infected honeybees) were fed syrup supplemented with 0.05% DMSO. According to the acute lethal dose (LD50) (62), the daily mean consumption of insecticides was below 1:194^th^ of the LD50 for coumaphos, thiamethoxam, and imidacloprid, and below 1:550^th^ and 1:150^th^ of the LD50 for low and high fipronil exposure, respectively (Fig. S1).

### Gut sampling and DNA purification

Honeybees were sacrificed 18 d after the initiation of experiments, but 15 d for the highest dose of fipronil due to high mortality, along with the corresponding controls. Regarding each replicate, the hindguts of 7 honeybees were dissected on ice, pooled, flash frozen in liquid nitrogen, and stored at –80°C. The presence or absence of *N. ceranae* spores was checked by microscopy (×400) before pooling. Samples were homogenized in 800 μL of RLT buffer (Qiagen) and ground using a microtube pestle. The mixture was transferred to a tube containing 0.150 g of 0.1-mm silica beads and cells were mechanically disrupted by a set of 6 bead-beating pulses of 30 s at 30 Hz, with 30-s interruptions (28). After 3 series of thermal shocks at 65°C and in liquid nitrogen, samples were centrifuged at 8,000×*g* for 5 min and 200 to 300 μL of the clear supernatant was transferred to a new tube, carefully avoiding both pelleted and floating slimy materials. One volume of ethanol containing 1% (v/v) of 2-mercaptoethanol was added to the sample, which was then transferred to a DNeasy spin column for DNA purification (DNeasy Mini Kit, Qiagen). DNA concentrations were quantified using the bisBenzimide DNA Quantitation Kit, Fluorescence Assay (Sigma-Aldrich).

### PCR quantification of bacterial taxa abundance

The theoretical specificity of primer pairs for the 16S rRNA gene was evaluated using the Testprime 1.0 tool (46) to estimate the coverage of bacterial taxa from the SILVA 132 database. Coverage was also estimated on honeybee microbiota 16S rRNA sequences from previous experiments (2, 21, 23, 59, 61, 83), requiring sequence assignment and selection. Taxonomic assignment was performed through a Blast search against the SILVA 132 database, keeping only sequences with >98% identity. Data reported by Corby-Harris *et al.* (21) were used to assign *Lactobacillus* sp. sequences to the Firm-4 and Firm-5 clades. Only sequences covering the whole amplicon were considered for each primer set, while sequences with ambiguous positions in the priming regions were removed. Primer sets have been experimentally tested for their ability to fulfill qPCR quality requirements (12) on honeybee gut material by checking their specificity (by gel electrophoresis), linear dynamic range, efficiency, and amplification reproducibility using 1:4 serial dilutions of gut DNA extracts.

qPCR was performed on a CFX96 Real-Time System Thermocycler (BioRad) in 96-well plates (Eurogentec RT-PL96-MQ) in a final volume of 20 μL containing 10 μL of 2X Absolute Blue qPCR SYBR Green Mix (Thermo Scientific), 10 pmol of each primer (Table 1), and 2 to 24 ng of total genomic DNA. The PCR program consisted of an initial step at 94°C for 10 min, and 40 cycles comprising 94°C for 25 s, 25 s of annealing at 53°C, and 25 s of elongation at 72°C. Treated and untreated samples from an identical replicate were always deposited on the same plate and all plates were duplicated. The specificity of the reactions was assessed by analyzing the melting curves of the amplified products (Bio-Rad CFX Manager software). The Cycle Quantification (C_Q_) values obtained were proportional to the log of input DNA amounts.

In contrast to previous studies, in which qPCR data were normalized to total DNA content or sample weights, we selected the total bacterial content as a reference. Therefore, we avoided biases due to non-bacterial DNA, *e.g*. DNA from *N. ceranae* or the honeybee, or those due to the loss of material following dissection or defecation. The total bacterial DNA content was estimated by the mean of C_Q_ values obtained with both the 341F/534R and BAC338F/BAC805R primer pairs (Table 1). It is important to note that the two pairs gave very similar C_Q_ values (correlation coefficient of 0.985; *n*=197). In each taxonomic group, the corresponding C_Q_ was subtracted from the mean reference C_Q_ of the same sample. Normalized C_Q_ were then compared pairwise. The log_2_ of the ratio of a taxonomic group under the treated condition relative to the untreated condition was obtained by subtracting the normalized C_Q_ of the control from the normalized C_Q_ of the treated sample from the same colony and sampling day. Statistical analyses were performed using PAST software (37).

## Results

Honeybees were exposed to the parasite *N. ceranae* or to low doses of the four neurotoxic insecticides. Daily sugar consumption was constant throughout the experiment and not significantly different between groups (Fig. S1). Survival analyses showed a significant decrease in honeybee survival for both winter and summer honeybees infected with *N. ceranae* or exposed to the highest dose of fipronil (Fig. S2). The four other insecticide treatments did not significantly reduce honeybee survival.

In order to assess the abundance of major bacterial taxa, published primer pairs targeting the 16S rRNA gene were checked for qPCR quality (Table 1). Ten primer pairs were eventually selected that target: total bacteria (two pairs), *Alphaproteobacteria*, *Bifidobacteriaceae* (almost exclusively represented by *Bifidobacterium* spp. in the honeybee gut), *Lactobacillus* spp. with one pair recognizing all *Lactobacillus* spp. (including Firm-4, Firm-5, and *L. kunkeei* clades) and another the *Lactobacillus* Firm-5 clade only, *Gammaproteobacteria*, *G. apicola* species, and *S. alvi* species (two pairs, including one recognizing *Neisseriaceae* that are almost exclusively represented by *S. alvi* in the honeybee microbiota). Principal component and ANOVA2 analyses suggested that season was the main factor explaining differences in the abundance of these taxa (Fig. S3). More than 79% of variance was explained by one principal component that appeared to be mainly linked to season and partly to treatments, particularly to *N. ceranae* infection.

**Fig. 1:**
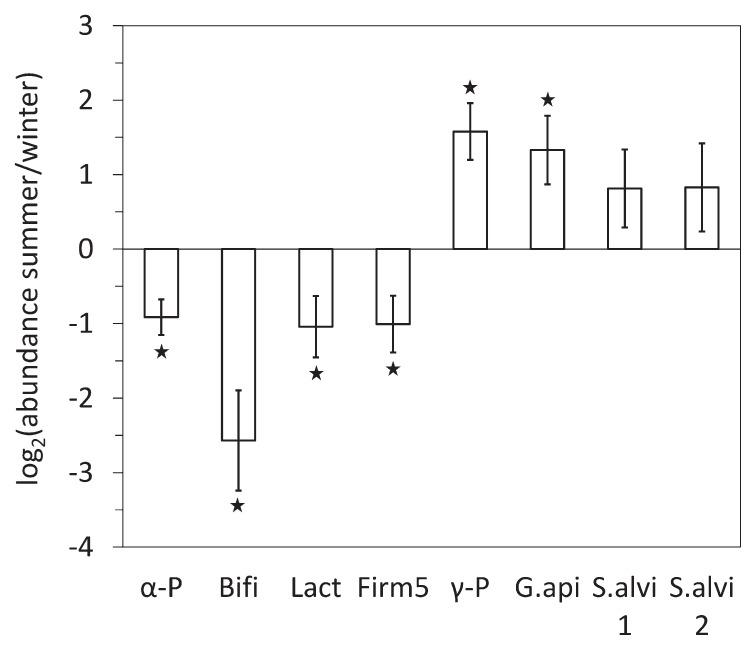
Comparison of the abundance of major bacterial taxa in the gut microbiota between summer and winter honeybees: *Alphaproteobacteria* (α-P), *Bifidobacterium* spp. (Bifi), *Lactobacillus* spp. (Lact), *Lactobacillus* Firm-5 clade only (Firm5), *Gammaproteobacteria* (γ-P), *Gilliamella apicola* (G.api), and *Snodgrassella alvi* (S.alvi1 and S.alvi2 using the primer pairs Neiss-F/Neiss-R and Beta-1009-qtF/Beta-1115-qtR, respectively). Interior worker honeybees were maintained for 18 d in cages (control conditions) before 16S rRNA gene quantification by qPCR in the hindgut. The y-axis represents the mean log_2_ of fold changes (FC) in taxon abundance between summer and winter workers. Positive and negative values denote higher and lower abundance, respectively, in summer bees than in winter bees. Data were gathered from five independent colony replicates. Bars represent 95% confidence intervals. Stars indicate significant differences (*P*<0.05) by a paired Wilcoxon signed-rank test.

The abundance of the major bacterial taxa was initially compared between summer and winter in untreated honeybees. *Bifidobacterium* spp., *Lactobacillus* spp., *Lactobacillus* Firm-5, and *Alphaproteobacteria* were significantly more abundant in winter, while *G. apicola* and *Gammaproteobacteria* were both more abundant in summer (Fig. 1). The core microbiota of the gut was subjected to global seasonal variations.

**Fig. 2:**
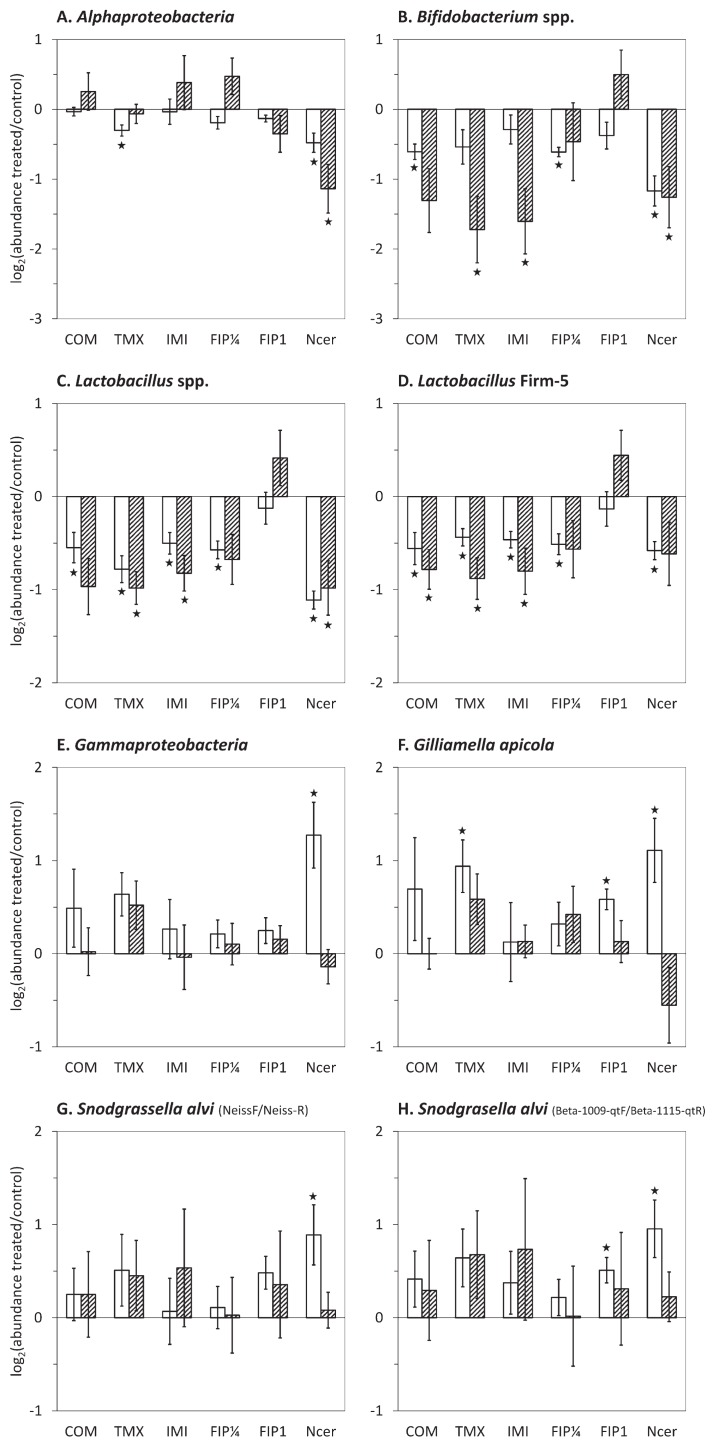
Abundance of major taxa in the gut microbiota of pesticide-exposed or *N. ceranae*-infected honeybees and untreated honeybees. Interior worker honeybees were submitted to a chronic exposure to 650 μg kg^−1^ coumaphos (COM), 1.7 μg kg^−1^ thiamethoxam (TMX), 3.5 μg kg^−1^ imidacloprid (IMI), 0.25 μg kg^−1^ (FIP¼), or 1 μg kg^−1^ fipronil (FIP1) or to an infection with *N. ceranae* (Ncer). The abundance of bacterial taxa was assessed by qPCR after 15 or 18 d in winter (white bars) and summer (hatched bars) honeybees. The y-axes depict the mean log_2_ of fold changes (FC) in abundance between treated and untreated samples. Negative and positive values denote lower and higher abundance, respectively, in response to stressors. Data were gathered from five independent colony replicates. Bars represent 95% confidence intervals. Stars indicate significant differences (*P*<0.05) by a paired Wilcoxon signed-rank test.

The abundance of bacterial groups in honeybees chronically exposed to fipronil, imidacloprid, thiamethoxam, or coumaphos was then compared to the untreated control (Fig. 2). Sublethal exposure to all insecticides induced a significant decrease in the relative abundance of *Lactobacillus* spp. in winter and summer honeybees, except for the treatment with the lower dose of fipronil (0.25 μg kg^−1^) for which the decrease was not significant in summer. The highest and lethal dose of fipronil (1 μg kg^−1^) did not significantly change *Lactobacillus* spp. abundance. Similar results were obtained with all *Lactobacillus* spp. or the Firm-5 clade only. A significant decrease in the abundance of *Bifidobacterium* spp. was observed in response to imidacloprid and thiamethoxam in summer honeybees and to coumaphos and the low dose of fipronil (0.25 μg kg^−1^) in winter honeybees. The abundance of *Bifidobacterium* spp. was not affected by the lethal dose of fipronil. Chronic exposure to insecticides induced other significant variations, but in winter honeybees only, with a decrease in the relative abundance of *Alphaproteobacteria* in response to thiamethoxam (Fig. 2A), an increase in *G. apicola* in response to thiamethoxam and 1 μg kg^−1^ fipronil (Fig. 2F), and an increase in *S. alvi* in response to 1 μg kg^−1^ fipronil using *S. alvi*-, but not *Neisseriaceae-*specific primers (Fig. 2G and H). Collectively, these results showed the similar effects of sublethal doses of insecticides that were more significant in winter honeybees.

*N. ceranae* infection induced variations in the proportion of all bacterial groups tested (Fig. 2). The abundance of *Alphaproteobacteria*, *Bifidobacterium* spp., and *Lactobacillus* spp. was significantly decreased by the parasite regardless of the season. In contrast, *Gammaproteobacteria*, *G. apicola*, and *S. alvi* significantly increased in winter honeybees only. However, one phylotype identified as *S. alvi* was less abundant in infected bees, as shown by Denaturating Gradient Gel Electrophoresis (Fig. S4), suggesting the complex rebalancing of *S. alvi* strains in infected honeybees.

## Discussion

### Seasonality of the honeybee microbiota and of stressor-induced variations

The abundance of the main bacterial groups of the gut microbiome differed in summer and winter honeybees (Fig. 1). The higher abundance of *G. apicola* during the beekeeping season was already suggested by Ludvigsen *et al.* (57). In contrast to previous studies (21, 57) seasonal dynamics were also observed for *Alphaproteobacteria*, *Bifidobacterium* spp., and *Lactobacillus* spp., which were less abundant in summer. Since the honeybee gut microbiota is influenced by the environmental landscape (42), it is logically influenced by season in temperate areas. Genomic data suggested that *G. apicola* participates in digestion through the breakdown of complex carbohydrates, such as the pectin contained in pollen (29, 52). The increased abundance of *G. apicola* in summer may be related to increases in these resources.

Responses to stressors also differed according to season (Fig. 2), with more significant results being observed in winter. The mortality of honeybees exposed to acute neonicotinoid treatments was previously reported to be higher in winter than in summer (7). We herein demonstrated that the microbiota, and, thus, the holobiont were more sensitive in winter. The lack of significance in summer bees may be related to a higher heterogeneity in microbial communities, reflected by the greater variance of data (Fig. 2 and S3) and higher α-diversity (57). These findings may be explained by the greater diversity of resources in summer (25, 42). Moreover, short-living summer bees have a more diverse physiology, engaging in the age-dependent division of labor with successive changes in the endocrine system (73), and microbiota richness and abundance have been suggested to differ with ontogenetic stages and behavioral tasks (40, 43). In contrast, in February, aged overwintering bees are more homogeneous, clustering inside the hive and feeding on less diverse reserves.

### Alterations in the honeybee microbiota by the intestinal parasite *N. ceranae*

Pathogens may alter the composition of the honeybee microbiota (30, 35), and the present results revealed that all major bacterial taxa were affected by *N. ceranae* (Fig. 2), with a decrease in lactic acid bacteria (LABs), including *Lactobacillus* spp. and *Bifidobacterium* spp., and an increase in *S. alvi* and *G. apicola*. The increase in *S. alvi* and *G. apicola* may have been due to a rebalance in the microbiota by occupying niches left vacant by LABs. The results of the DGGE analysis suggested that while all *S. alvi* species experienced an increase in abundance, the content of a specific strain decreased. This opposite variation in strain content was previously reported for *G. apicola* in response to an antibiotic treatment, suggesting variations in the ability of strains to cope with stress (66). This important dysbiosis was not observed in *A. mellifera* in previous studies (41, 54, 58). In the Asian honeybee *A. cerana*, Li *et al.* (53) observed a similar decrease in the abundance of *Bifidobacterium* spp., but no significant change in *Lactobacillus* spp..

It is unlikely that *N. ceranae* directly affects gut microbial communities because its development entirely occurs within midgut epithelial cells. However, it may alter honeybee midgut homeostasis. *N. ceranae* induces a degeneration in the midgut epithelium and peritrophic matrix (26, 39, 48) that may alter the activation of the immune system by luminal bacteria (38, 47). The infection also up-regulates the antioxidant system, reduces the production of reactive oxygen species (ROS) (26, 65, 81, 82), and may provoke immunosuppression by inhibiting the production of antimicrobial peptides (AMP) (3, 5, 16, 55). No data are available on the honeybee hindgut. Interestingly, the microsporidian parasite *Paranosema locustae* alters the gut microbiota of *Locusta migratoria*, but also induces acidification and an increase in ROS in the hindgut (74, 78). By affecting the gut ecosystem, possibly through changes in AMPs, ROS, or pH, *N. ceranae* may favor or impair bacterial communities.

### Alterations in the honeybee gut microbiota by insecticides

Honeybees were chronically exposed to concentrations of insecticides that correspond to residue levels detected in hive matrices (18, 45, 51, 62), denoting realistic exposure. These treatments led to variations in the honeybee core microbiota taxa, with a general decrease in LABs (Fig. 2). Kakumanu *et al.* (44) also observed a significant decrease in *Lactobacillus* spp. in response to the fungicide chlorothalonil, but a slight increase in *Bifidobacterium* spp. in response to coumaphos. This opposite variation in *Bifidobacterium* spp. suggests the strong influence of the mode of exposure (oral exposure in cages *vs* an in-hive CheckMite+® treatment) on microbial responses. In contrast to our results, Raymann *et al.* (68) reported that imidacloprid did not significantly affect the microbiota. The treatment with 1.0 μg kg^−1^ fipronil did not have a stronger effect on LABs than the 0.25 μg kg^−1^ treatment, but induced an increase in *G. apicola* and *S. alvi* in winter (Fig. 2). This higher insecticide concentration was lethal (Fig. S2) and may have had a strong effect on morbidity in the microbiota because dying honeybees were sampled.

Insecticides may have a direct impact on bacterial growth that strongly varies upon insecticides and bacterial strains (75). Thus, insecticides from different families may not have the same direct impact on gut bacteria. The similar effects observed in response to different insecticides (Fig. 2) may have been due to aspecific changes in the gut homeostasis that may secondarily affect the microbiota. In the midgut, chronic and oral exposure to insecticides altered the immune system, activated antioxidant defenses, and induced damage in epithelial cells (5, 9, 15, 20, 31, 68). Thus, gut bacterial communities may have to cope with changes in their environment.

Ingested neurotoxic insecticides are absorbed in the gut and diffuse throughout the whole insect body (76), eventually affecting the nervous system (14). Neonicotinoids, such as thiamethoxam and imidacloprid, induce morphological, cytological, and transcriptomic alterations in the brain and disrupt the hormonal balance (20). Thus, besides local effects on the gut, insecticides exert pleiotropic effects that may, in turn, affect the gut and its microbiota, which is considered to be connected to the nervous system in mammals (69). This indirect impact on the gut has been demonstrated based on changes in midgut antioxidant activities following a single topical exposure to fipronil (13).

### Dysbiosis may sensitize the honeybee to other stressors

The microbiota protects the host against pathogens by stimulating the immune system and providing competition for niche occupation (36). The honeybee gut microbiota is considered to induce AMP production and the LABs themselves exhibit antibacterial properties (50, 67, 80). The suppression of honeybee gut bacteria by antibiotics increased honeybee vulnerability to *N. ceranae* (54), and some bacteria, including LABs, may conversely reduce parasite proliferation (22, 27). In contrast, feeding supplemented with *S. alvi* increased honeybee sensitivity to the trypanosomal parasite *Lotmaria passim* (72).

The insect microbiota may also comprise strains with the ability to enhance host resistance to insecticides (84). In the moth *Plutella xylostella*, a positive correlation was observed between *Lactobacillales* abundance and fipronil resistance. Moreover, some gut bacteria isolated from insects, including *Lactobacillus* spp., are considered to metabolize insecticides (1, 24). Thus, dysbiosis and a reduction in LABs may sensitize the honeybee to pathogens and pesticides.

The season-dependent gut microbiota may be regarded as a modulator of interactions between the honeybee and its stressors, emphasizing the need to consider the holobiont, *i.e*. the insect and its symbionts, both in summer and winter, when assessing the effects of stressors.

## SUPPLEMENTARY MATERIAL



## Figures and Tables

**Table 1:** Primer pairs tested for the QPCR quantification of the honeybee microbiota major bacterial taxa.

Primer pairs and sequences (5′→3′)	Targeted group and % coverage in SILVA database (in honeybee microbiota)	Amplicon size (bp)	Linearity (r^2^) Efficiency (E)	validated DNA range^a^ and C_Q_ range	Reference
***Bacteria***					
341F CCTACGGGAGGCAGCAG *	*Bacteria* 81.7	169–195	r^2^ = 0,999	50 pg to 50 ng	11
534R ATTACCGCGGCTGCTGGCA *			E = 0,92	C_Q_ 10 to 22	
BAC338F ACTCCTACGGGAGGCAG *	*Bacteria* 77.4	443–469	r^2^ = 0,999	50 pg to 10 ng^b^	86
BAC805R GACTACCAGGGTATCTAATCC *			E = 0,85	C_Q_ 13.5 to 23	
BACT1369F CGGTGAATACGTTCYCGG	*Bacteria* 25.4	133–146	r^2^ = 0,999	50 pg to 50 ng	77
PROK1492R GGWTACCTTGTTACGACTT			E = 0,84	C_Q_ 11 to 23	
926F AAACTCAAAKGAATTGACGG	*Bacteria* 78.4	172–179	r^2^ = 0,999	50 pg to 50 ng	6
1062R CTCACRRCACGAGCTGAC			E = 0,87	C_Q_ 11 to 22	
***Bifidobacterium* spp**.					
Bifi-F2 TCGCGTCYGGTGTGAAAG *	*Bifidobacteriaceae* 89.3	243	r^2^ = 0,999	50 pg to 50 ng	70
Bifi-R2 CCACATCCAGCRTCCAC *	*Bifidobacterium* spp. 89.0 (96.1, *n*=51)		E = 0,78	C_Q_ 14.5 to 25	
Bifi-F1 CAAGCGAGAGTGAGTGTACC	*Bifidobacterium* spp. 2.5 (88.7, *n*=53)	165	r^2^ = 0,999	50 pg to 50 ng	53
Bifi-R1 GCCGATCCACCGTTAAGC			E = 0,90	C_Q_ 14.5 to 25.5	
Act920F3 TACGGCCGCAAGGCTA	*Actinobacteria* 86.7	299–307	r^2^ = 0,999	not validated	6
Act1200R TCRTCCCCACCTTCCTCCG	*Bifidobacteriaceae* 84.8		E = 0,67		
	*Bifidobacterium* spp. 86.5 (94.2, *n*=52)				
***Lactobacillus* spp**.					
Lact-F1 TAACGCATTAAGCACTCC *	*Lactobacillus* 3 (28.6, *n*=1,705)	270	r^2^ = 0,998	50 pg to 50 ng	53
Lact-R1 GCTGGCAACTAATAATAAGG *	*L. kunkeei* (0, *n*=875)		E = 0,89	C_Q_ 13.5 to 24.5	
	Firm-4 (0, *n*=88)				
	Firm-5 (97.2, *n*=424)				
Lact-F2 AGCAGTAGGGAATCTTCCA *	*Lactobacillus* 87.2 (95.7, *n*=1,690)	341	r^2^ = 0,997	50 pg to 10 ng^b^	70
Lact-R2 CACCGCTACACATGGAG *	*L. kunkeei* (96.8, *n*=875)		E = 1,08	C_Q_ 14 to 23	
	Firm-4 (97.7, *n*=88)				
	Firm-5 (91.7, *n*=424)				
928F-Firm TGAAACTYAAAGGAATTGACG	*Firmicutes* 73.7	149–157	r^2^ = 0,998	50 pg to 10 ng^b^	6
1040FirmR ACCATGCACCACCTGTC	*Lactobacillales* 73.3		E = 0,83	C_Q_ 14.5 to 23.5	
	*Lactobacillus* (43.6) (69.1, *n*=1,705)				
	*L. kunkeei* (97.7-8, *n*=875)				
	Firm-4 (97.7, *n*=88)				
	Firm-5 (0.5, *n*=424)				
	*Mollicutes* 39.8				
***Alphaproteobacteria***					
a682F CDAGTGTAGAGGTGAAATT *	*Alphaproteobacteria* 63.6	245–250	r^2^ = 0,998	50 pg to 50 ng	6
908aR CCCCGTCAATTCCTTTGAGTT *	*Bartonella* 25.4 (89.6, *n*=77)		E = 0,91	C_Q_ 14.5 to 25	
	*Acetobacteraceae* 0.8 (10.0, *n*=201)				
***Gammaproteobacteria***					
1080γF TCGTCAGCTCGTGTYGTGA *	*Gammaproteobacteria* 61.2	146–160	r^2^ = 0,999	50 pg to 50 ng	6
γ1202R CGTAAGGGCCATGATG *	*Gilliamella* 96.9 (97.1, *n*=516)		E = 0,90	C_Q_ 14.5 to 25	
	*Snodgrasella* 0 (0, *n*=342)				
	*Enterobacteriales* 94.0				
***Snodgrasella alvi***					
Neiss-F AAGCGGTGGATGATGTGG *	*Neisseriaceae* 79.4	194–199	r^2^ = 0,999	50 pg to 50 ng	53
Neiss-R TGATGGCAACTAATGACAAGG *	*Snodgrassella* 86.4 (95.1, *n*=344)		E = 0,90	C_Q_ 13.5 to 23.5	
	*Snodgrassella alvi* 100 (95.6, *n*=226)				
Beta-1009-qtF CTTAGAGATAGGAGAGTG *	*Snodgrasella* 77.3 (93.0, *n*=344)	127–128	r^2^ = 0,999	50 pg to 50 ng	60
Beta-1115-qtR AATGATGGCAACTAATGACAA *	*Snodgrassella alvi* 76.9 (94.2, *n*=226)		E = 0,87	C_Q_ 15 to 24	
Snod-F GGAATTTCTTAGAGATAGGAAAGTG	*Snodgrasella* 11.4 (0, *n*=344)	136	r^2^ = 0,999	50 pg to 50 ng	85
Snod-R TTAATGATGGCAACTAATGACAA	*Snodgrassella alvi* 23.1 (0, *n*=226)		E = 0,86	C_Q_ 15 to 26	
***Gilliamella apicola***					
G1-459-qtF GTATCTAATAGGTGCATCAATT *	*Gilliamella* 23.0 (71.6, *n*=232)	210	r^2^ = 0,999	50 pg to 50 ng	60
G1-648-qtR TCCTCTACAATACTCTAGTT *	*Gilliamella apícola* 22.9 (80.7, *n*=109)		E = 0,91	C_Q_ 14.5 to 25.5	
Gill-F CCTTTGTTGCCATCGATTAGG	*Gilliamella* 28.0 (1.7, *n*=355)	249	r^2^ = 0,999	50 pg to 50 ng	85
Gill-R GACATTCTGATTCACGATTACTAGC	*Gilliamella apícola* 37.5 (0.9, *n*=233)		E = 0,83	C_Q_ 16.5 to 28	
Past-F TTGTTGCCAGCGATTAGG	*Gilliamella apícola* 4.2 (2.1, *n*=233)	243	r^2^ = 0,998	200 pg to 50 ng^c^	53
Past-R ATTCTGATTCACGATTACTAGC	*Frischella perrara* 100 (94.9, *n*=59)		E = 0,75	C_Q_ 18 to 27.5	
***Bacteroidetes***					
798cfbF CRAACAGGATTAGATACCCT	*Bacteroidia* 86.2 (100, *n*=44)	202–208	r^2^ = 0,998	50 pg to 50 ng	6
cfb967R GGTAAGGTTCCTCGCGTAT	*Spirochaetaceae* 42.8		E = 0,90	C_Q_ 21.5 to 33 d	

a total DNA from the honeybee gut

b not quantitative for higher DNA concentrations

c not quantitative for lower DNA concentrations

d high C_Q_ precluded any analyses

Selected pairs are indicated by an *.
