# Supplementary material for: The Honeybee Gut Microbiota Is Altered after Chronic Exposure to Different Families of Insecticides and Infection by Nosema ceranae
Source: Microbes Environ. 2019 Aug 3;34(3):226–33. doi: 10.1264/jsme2.ME18169 (PMC6759349; doi:10.1264/jsme2.ME18169)
Supplement: Supplementary file 1 [file 34_226_s1.pdf]

A.

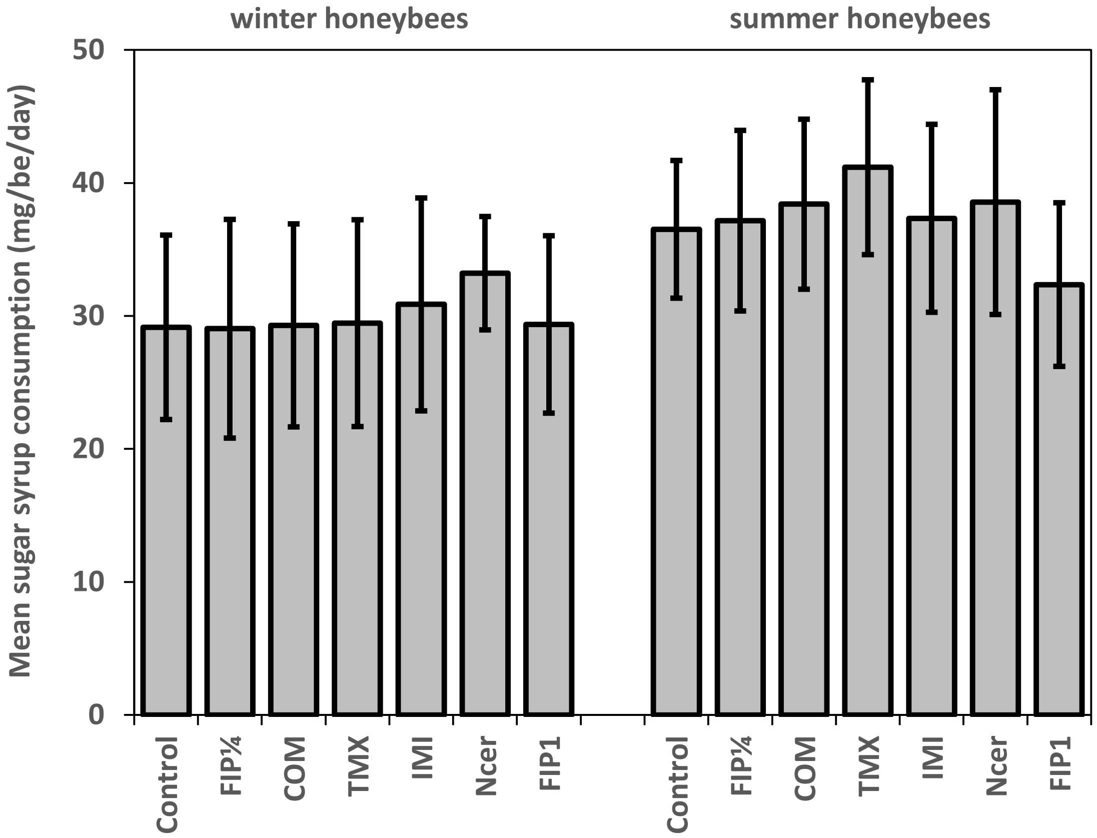

B.

Condition		FIP¼<br>fipronil<br>0.25 ng/g	COM<br>coumaphos<br>650 ng/g	TMX<br>thiamethoxam<br>1.7 ng/g	IMI<br>imidacloprid<br>3.5 ng/g	FIP1<br>fipronil<br>1.0 ng/g
Consumption (ng/bee/day) ± IC95	Winter honeybees	0.0074<br>± 0.0003	20.69<br>± 0.87	0.053<br>± 0.002	0.114<br>± 0.003	0.030<br>± 0.002
	Summer honeybees	0.0089<br>± 0.0006	23.68<br>± 1.51	0.067<br>± 0.004	0.127<br>± 0.006	0.030<br>± 0.002
LD50 (ng/bee)		5	4630	15	28	5
Consumption (fraction of LD50/bee/day)	Winter honeybees	1:679	1:224	1:282	1:245	1:167
	Summer honeybees	1:560	1:194	1:222	1:221	1:164

Supplementary Figure S1. Sucrose (A) and insecticide (B) consumption of honeybees chronically exposed to insecticides or infected with *Nosema ceranae*. Insecticides were added in the feeding sugar syrup *ad. lib.*, and spores of *N. ceranae* were orally administrated to the honeybees at the beginning of the experiment. Control bees were not exposed to any stressor. (**A**) Mean daily sugar consumption in winter (left panel) and summer (right panel) honeybees. Bars represent the 95% confidence intervals. No significant difference was observed. (**B**) Mean daily consumption of insecticides and comparison with the oral LD50 given by Mullin *et al*. (62).

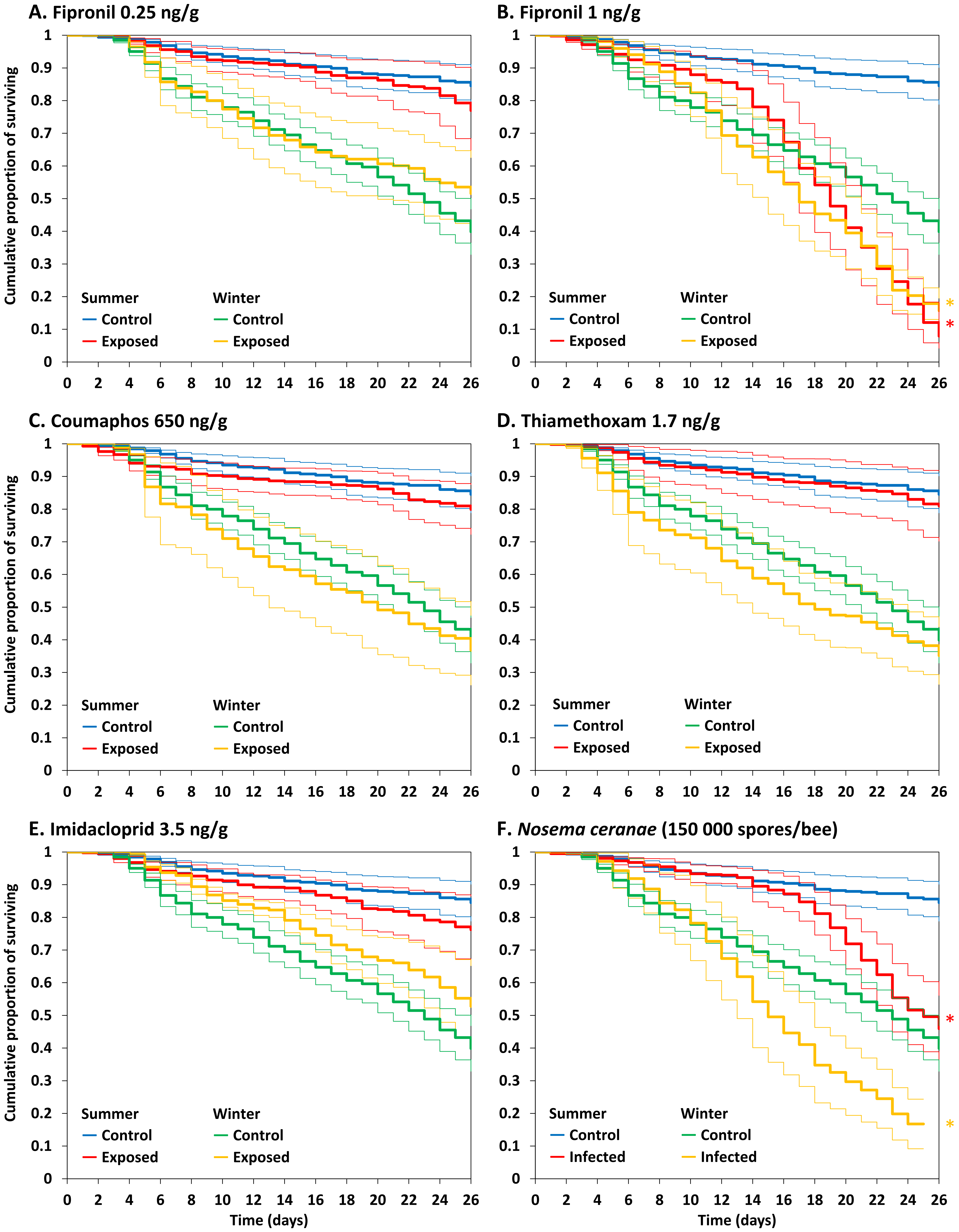

Supplementary Figure S2. Cumulative proportion of surviving honeybees chronically exposed to insecticides (A-E) or infected with spores of *Nosema ceranae* (F) in winter (red) or summer (yellow) compared to untreated control in winter (blue) or summer (green). Insecticides were added in the feeding sugar syrup, and spores of *N. ceranae* were orally administrated at the beginning of the experiment. Control bees were not exposed to any stressor. Survival proportion was estimated using the Kaplan-Meier method. Thick curves represent the mean values from five colony replicates (n = 5 with 66 to 74 bees per replicate and per condition at day 0) and the thin curves represent the amplitude of the standard error. Log rank χ^2 tests using data from single colony replicates showed significant effects (p<0.005) of 0.25 ng/g fipronil on one colony in winter, of coumaphos on one colony in summer, and of imidacloprid on two colonies in summer. As these effects were clearly not reproduced among replicates, they were considered as not significant. Only the decrease of survival in infected bees (F) and in bees submitted to 1 ng/g fipronil (B) was significant in all colony replicates (with χ^2>9.1 and p<0.0025), and thus considered as globally significant (*).

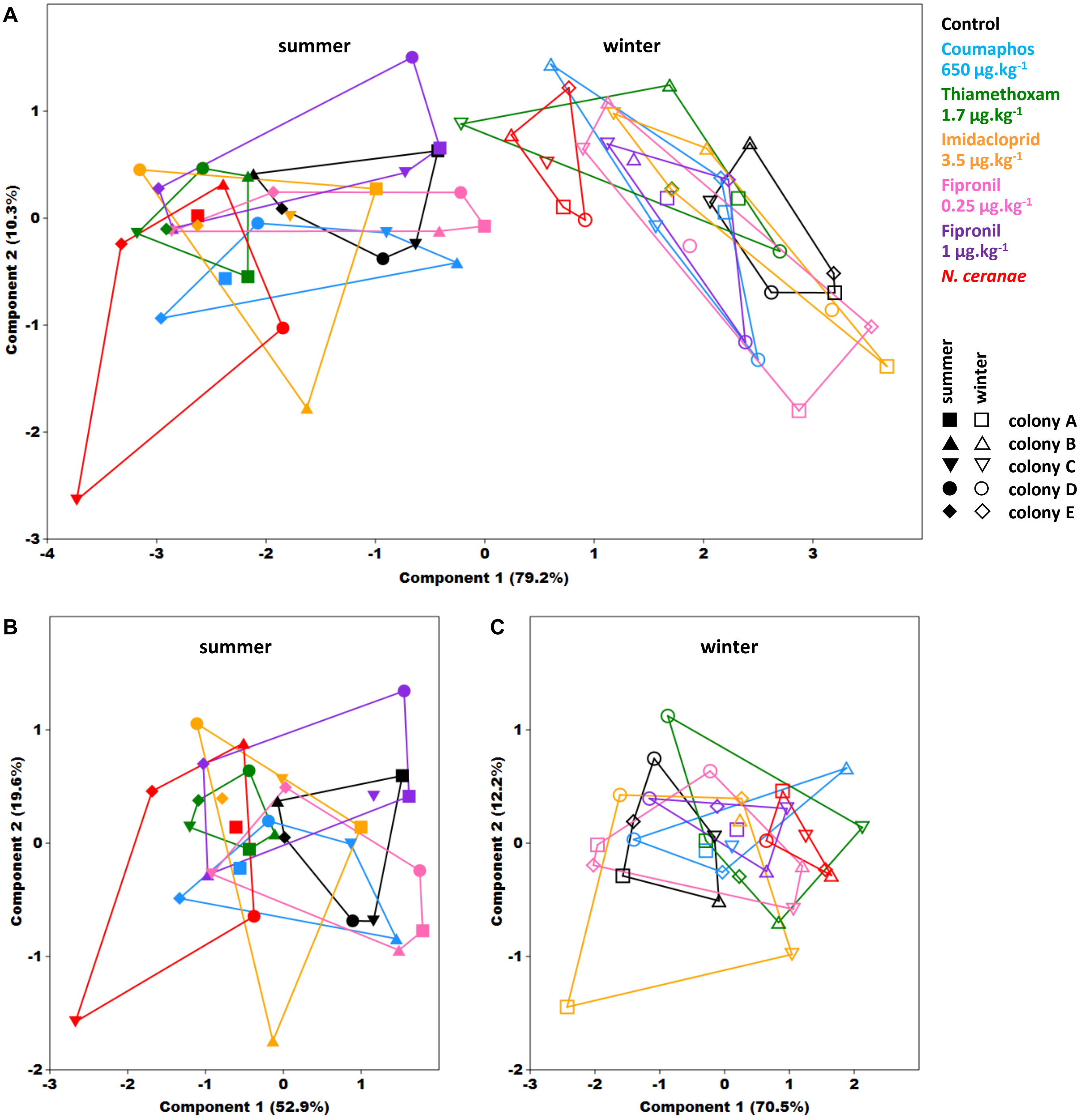

Supplementary Figure S3. Principal component analysis of QPCR data. Analyses were performed using the normalized Cq to total bacterial content in all **(A)** or in summer (filled signs, **B**) or in winter (empty signs, **C**) honeybees. In order to avoid redundant data for the same taxa, only values obtained for all *Lactobacillus* spp., *Bifidobacterium* spp., *Alphaproteobacteria*, *G. apicola* and *S. alvi* were included. Almost identical results were obtained exchanging primer pairs data for a similar taxon. In **A** more than 79% of the variance explained by one principal component that seemed mainly linked to the season. In **B** and **C**, only control and *N. ceranae* observations were clearly separated. In all analyses, only the first component was significant (broken stick model). Two-ways ANOVA (not shown) revealed significant effect of season for all taxa except *Neisseriaceae* and *S. alvi* (in accordance with Fig.1), and significant effect of treatment in accordance with the Fig. 2.

A. ***Bacteria*** **B.** ***Neisseriaceae***

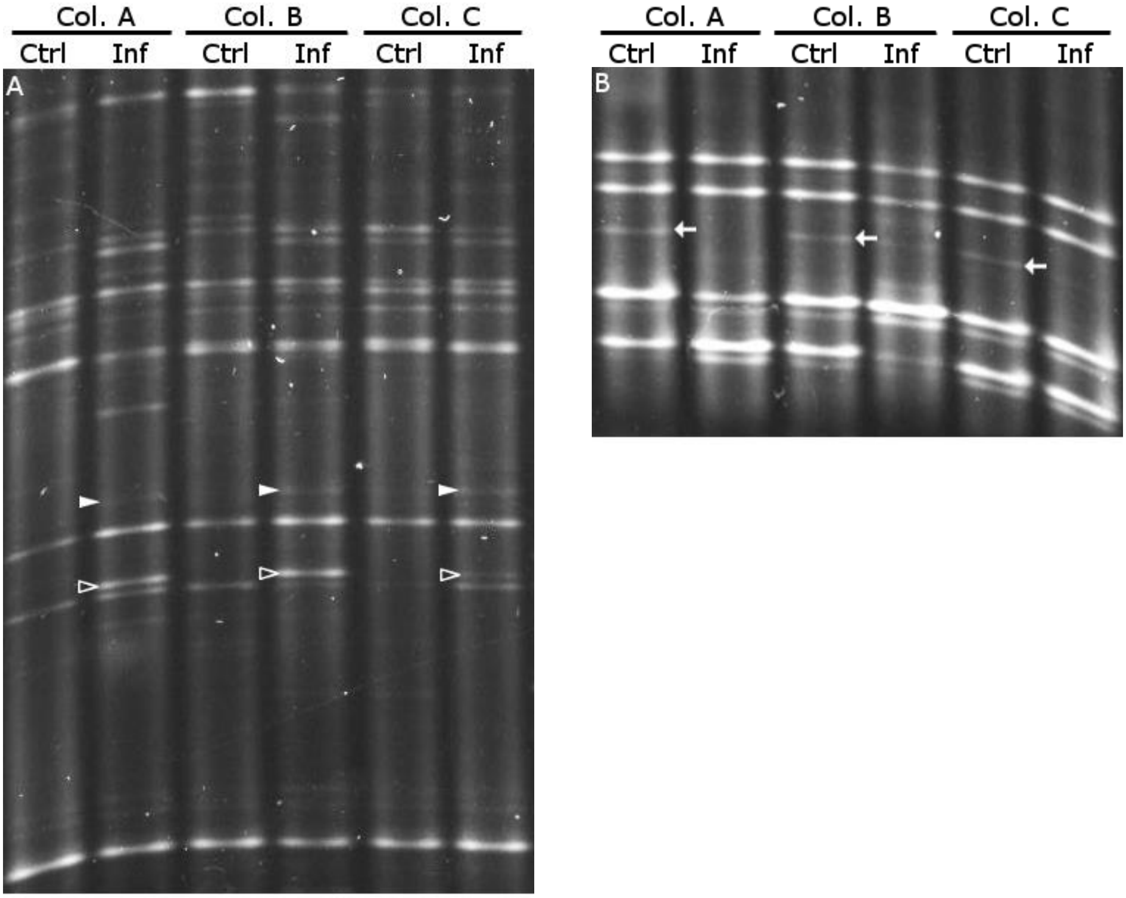

Supplementary Figure S4. Denaturing Gradient Gel Electrophoresis (DGGE) profiles of total bacterial (A) and *Neisseriaceae*-specific (B) 16S rDNA fragments present in the gut microbiota from uninfected and *N. ceranae*-infected honeybees. Winter honeybees from three colonies (Col. A, Col. B, Col. C) were infected with *N. ceranae* spores (Inf.) or not infected (Ctrl). 5 or 6 bees per condition and per replicate were sacrificed after 22 days. Their gut was dissected and the total DNA was extracted as described in the main article. The V3-V5 region of the bacterial 16S rRNA gene was amplified using GC-338F (5'-CGCCCGCCGCGCGCGGCGGGCGGGGCGGGGGCACGGA-CTCCTACGGGAGGCAGCAG-3') and 518R (5’-ATTACCGCGGCTGCTGG-3’) primers (3) following PCR conditions described by Mrázek *et al.* (2). The V6 region of *Neisseiriaceae*, including *S. alvi*, 16S rRNA gene was amplified using GC-968F (5’-CGCCCGGGGCGCGCGCCGGGCGGGGCGGGGGCACGGGGGGAACGCGAAGAACCTTAC-3’ from Crouzet *et al.* (1)) and Beta-1115-qtR (Table 1) primers following PCR conditions described by Crouzet *et al.* (1). 300 ng of amplification products were separated on 9% polyacrylamide gels with 35–60 % denaturing gradient of 7 M urea and 40 % formamide (V/V) as in Mrázek *et al.* (2). Gels were stained using GelStar dye (Lonza) and visualized on UV light on UV Biospectrum® Imaging System™ (UVP). Nine bands of interest (arrows) were excised from the gel and cloned into pGEM®-T Vector (Promega). The inserts of 3 or 4 clones per band were sequenced (Eurofins Genomics). Two bacterial phylotypes **(A)** were associated to *N. ceranae* infection (empty and filled triangles). Their identification was not possible as they were mixtures of several species including *S. alvi*, *Lactobacillus spp.*, *Providencia spp.*, *Bartonella spp.*, *G. apicola* and *F. perrara*. The neisseriacean phylotype (arrows in **B**) was attributed to *S. alvi* (>80% of obtained sequences with >99% identity, accession number MH936011) and was associated with uninfected bees (arrow) in the three colony-replicates.

References :

1. Crouzet, O., I. Batisson, P. Besse-Hoggan, *et al*. 2010. Response of soil microbial communities to the herbicide mesotrione: a dose-effect microcosm approach. Soil Biol. Biochem. 42:193–202.
2. Mrázek, J., L. Štrosová, K. Fliegerová, *et al*. 2008. Diversity of insect intestinal microflora. Folia Microbiol. 53:229–233.
3. Muyzer, G. 1993. Profiling of complex microbial populations by Denaturing Gradient Gel Electrophoresis analysis of Polymerase Chain Reaction-amplified genes coding for 16S rRNA. Appl. Environ. Microbiol. 59:695–700.
